# A probabilistic computation framework to estimate the dawn phenomenon in type 2 diabetes using continuous glucose monitoring

**DOI:** 10.1038/s41598-024-52461-1

**Published:** 2024-02-05

**Authors:** Souptik Barua, Namino Glantz, Arianna Larez, Wendy Bevier, Ashutosh Sabharwal, David Kerr

**Affiliations:** 1 Division of Precision Medicine, Department of Medicine, New York University Grossman School of Medicine, New York, NY USA; 2 Department of Electrical and Computer Engineering, Rice University, Houston, TX USA; 3 Sansum Diabetes Research Institute, Santa Barbara, CA USA; 4 Santa Barbara County Education Office, Santa Barbara, CA USA; 5 Center for Health Systems Research, Sutter Health, Santa Barbara, CA USA

**Keywords:** Computational models, Statistical methods, Diabetes

## Abstract

In type 2 diabetes (T2D), the dawn phenomenon is an overnight glucose rise recognized to contribute to overall glycemia and is a potential target for therapeutic intervention. Existing CGM-based approaches do not account for sensor error, which can mask the true extent of the dawn phenomenon. To address this challenge, we developed a probabilistic framework that incorporates sensor error to assign a probability to the occurrence of dawn phenomenon. In contrast, the current approaches label glucose fluctuations as dawn phenomena as a binary yes/no. We compared the proposed probabilistic model with a standard binary model on CGM data from 173 participants (71% female, 87% Hispanic/Latino, 54 ± 12 years, with either a diagnosis of T2D for six months or with an elevated risk of T2D) stratified by HbA_1c_ levels into normal but at risk for T2D, with pre-T2D, or with non-insulin-treated T2D. The probabilistic model revealed a higher dawn phenomenon frequency in T2D [49% (95% CI 37–63%)] compared to pre-T2D [36% (95% CI 31–48%), p = 0.01] and at-risk participants [34% (95% CI 27–39%), p < 0.0001]. While these trends were also found using the binary approach, the probabilistic model identified significantly greater dawn phenomenon frequency than the traditional binary model across all three HbA_1c_ sub-groups (p < 0.0001), indicating its potential to detect the dawn phenomenon earlier across diabetes risk categories.

## Introduction

The dawn phenomenon refers to abnormally high glucose rise in the early morning hours before breakfast in the absence of nocturnal hypoglycemia. First described in 1981, the dawn phenomenon has been found in type 1 (T1D) and type 2 diabetes (T2D), in prediabetes, and in individuals with otherwise normal glucose tolerance^1–3^. The early morning transient hyperglycemia appears to be due to an increase in glycogenolysis and gluconeogenesis associated with inadequate insulin secretion, sub-optimal insulin action, or insulin resistance. The excess hepatic glucose production appears to be a consequence of the unopposed action of growth hormone and other counter-regulatory hormones^4,5^. The dawn phenomenon has been reported in more than 50% of adults with T2D, contributing to an average rise of 0.4% in HbA_1c_ levels^3,6^. The presence of the dawn phenomenon in T2D remains a therapeutic challenge^6^.

With the increasing availability and accuracy of continuous glucose monitoring (CGM) systems, recent studies have quantified the dawn phenomenon in participants with T1D and T2D and those with impaired glucose regulation^6–11^. However, currently, there is no consensus definition of the dawn phenomenon using CGM. As the dawn glucose rise from nocturnal nadir to pre-breakfast time is subject to large day-to-day fluctuations, Monnier et al*.* first defined the dawn phenomenon in T2D based on CGM profiles as a rise in glucose levels of 20 mg/dL or more from the nocturnal glucose nadir to the pre-breakfast glucose level^6^. In T1D, others have suggested a less stringent threshold of 10 mg/dL^11^. Overall, a binary determination, i.e., dawn phenomenon has either happened or not happened, based on a pre-determined glucose threshold does not account for *CGM* *measurement* *errors* in the dawn phenomenon evaluation. The reported error metrics of common CGMs such as the Abbott Freestyle Libre Pro^12^ and the Dexcom G6^13^ can have a non-trivial impact on detecting small glucose changes, as the error variance of these devices can be 10–20 mg/dL, which is of the same order of magnitude as the dawn phenomena. Thus, in order to use CGMs to identify dawn phenomena reliably, we need a statistically sound method to account for CGM measurement error in assessing dawn phenomenon from CGM traces.

This study proposes a novel approach for estimating the dawn phenomenon that accounts for CGM errors. Our method assigns a *probability* that the dawn phenomenon has occurred rather than a binary determination. We piloted this approach by examining the dawn phenomenon in predominantly Hispanic/Latino adults, a population with a known disproportionate burden of T2D, as the contribution of the dawn phenomenon to overall dysglycemia in this population remains unclear.

## Results

### Participant cohort

A total of 189 participants completed two weeks of CGM wear. However, only 173 participants had at least the recommended ten days of usable CGM data^14^ and were included in subsequent analyses. Demographic and clinical details of the primary cohort of 173 participants are presented in Table 1**.** Participants were stratified by baseline HbA_1c_ levels into at-risk (HbA_1c_ < 5.7%, n = 64), pre-T2D (5.7% ≤ HbA_1c_ ≤ 6.4%, n = 57), and T2D (HbA_1c_ > 6.4%, n = 52) as per the American Diabetes Association guidelines^15^.

**Table 1:** Demographic and clinical measurements for the participant cohort.

Variable	Whole cohort	By baseline HbA_1c_		
		At-risk (HbA_1c_ < 5.7%)	Pre-T2D (5.7% ≤ HbA_1c_ ≤ 6.4%)	HbA_1c_ > 6.4%
Number of participants	173	64	57	52
Age	54.3 ± 12.2	49.8 ± 12.3	58.0 ± 10.6	55.8 ± 12.2
Gender	123 female 50 male	43 female 21 male	47 female 10 male	33 female 19 male
BMI (kg/m^2^)	30.5 ± 5.3	29.9 ± 5.4	29.7 ± 4.0	33.2 ± 6.2
Waist circumference (cm)	98.4 ± 11.9	95.9 ± 12.0	96.2 ± 10.2	103.7 ± 12.1
Hispanic/Latino	151 (87.3%)	54 (84.4%)	52 (91.2%)	45 (86.5%)
Born in Mexico	153 (88.4%)	60 (93.8%)	50 (87.7%)	43 (82.7%)
HbA_1c_ (%)	6.4 ± 1.5	5.4 ± 0.2	6.0 ± 0.2	8.1 ± 1.8
Medication status*				
Not on medication	49 (28.3%)	27 (42.2%)	14 (24.6%)	8 (15.4%)
On medication Metformin	124 (71.7%) 22	37 (57.8%) 3	43 (75.4%) 6	44 (84.6%) 13

Values for age, BMI, waist circumference, and HbA_1c_ reported as mean ± SD.

*T2D* type 2 diabetes, *BMI* body mass index.

*Some participants were on only one type of medication while others were on multiple types of medication.

### Dawn phenomenon analysis

#### Quantifying the occurrence of dawn phenomenon: binary vs probabilistic model

**Figure 1:**
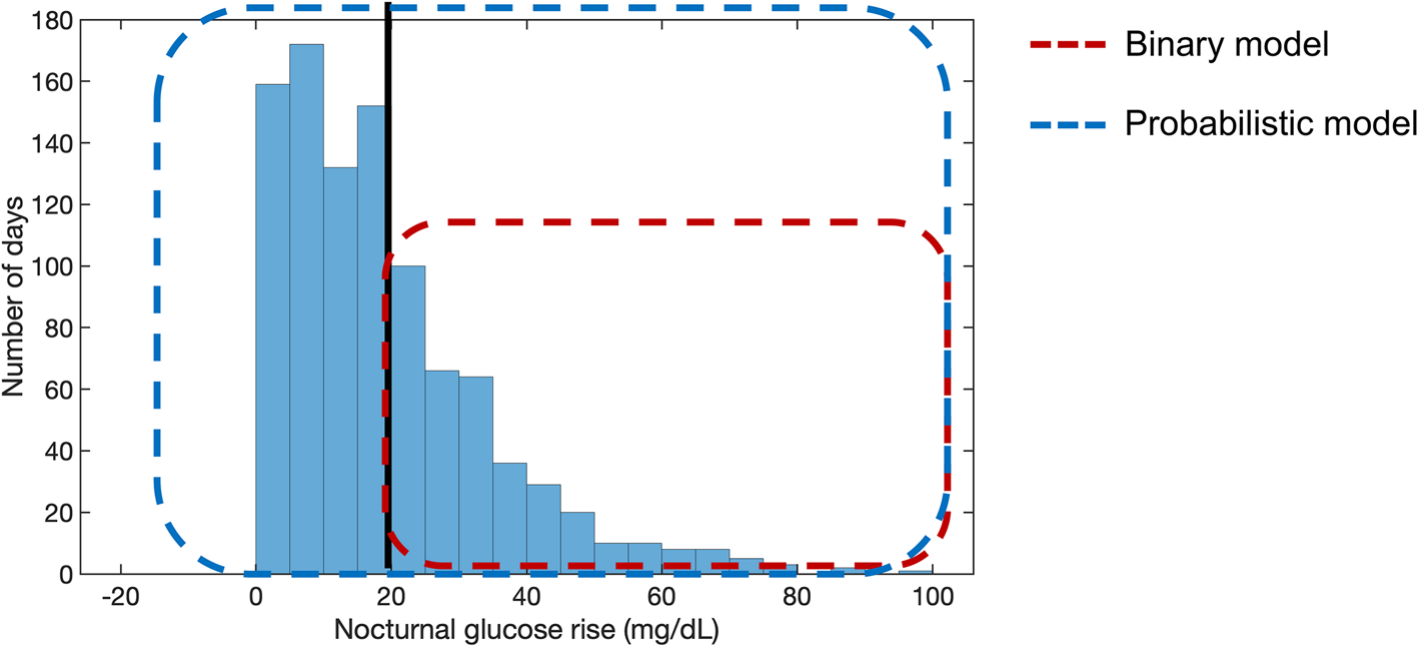
Histogram showing how frequently different values of nocturnal glucose rise occurred for the whole participant cohort (n = 173). The red dotted area represents the days considered by the standard binary model for dawn phenomenon analysis. The blue dotted area represents the days considered by the proposed probabilistic model for the dawn phenomenon analysis. Our approach accounts for CGM error and therefore includes the contribution of all days in the analysis weighted by an appropriate probability value.

CGM data from 173 participants with an average of 14.6 ± 1.8 days of data per participant were analyzed. A total of 2631 days of CGM data were available, out of which 978 days had a valid breakfast glucose peak and were used for all subsequent computational analysis. We first computed a histogram of nocturnal glucose rise across the 978 valid days of CGM data (Fig. 1). 615 days (62.7%) had a glucose rise of less than 20 mg/dL, while 363 days (37.3%) had a glucose rise greater than or equal to 20 mg/dL. The binary approach, which does not account for CGM error, therefore identifies 363 days as dawn phenomenon while ignoring the remaining days. On the other hand, the proposed probabilistic approach accounts for CGM error by including all 978 days in the dawn phenomenon estimation weighted by a probability value. Adding the probability of dawn phenomenon for each of the 978 days, we obtain an effective 431 days of dawn phenomenon. Nearly a third of the 431 days (158 days, 36.6%) came from the days with measured nocturnal glucose rises *less than* 20 mg/dL. While 367 days had nocturnal glucose rise greater than 20 mg/dL, after appropriate weighting by the probabilistic model, those days effectively contributed 273 days (63.4%) of dawn phenomenon. This result highlights two important features of the probabilistic approach. First, instead of ignoring the days with measured nocturnal glucose rises *less* than 20 mg/dL, the probabilistic model includes them with appropriate weighting. Second, instead of giving equal and 100% weight to days with measured nocturnal glucose rises *greater* than 20 mg/dL, the model weights a given day based on how much higher than 20 mg/dl the rise was. As an example: for two days with measured nocturnal glucose elevations of 25 and 50 mg/dL, the binary model considers the dawn phenomenon to have occurred with equal likelihood (100%) on both days, while the probabilistic model assigns a higher probability to the latter because it is more likely to have had a true > 20 mg/dL nocturnal glucose rise.

#### Dawn phenomenon metrics by HbA_1c_ sub-groups

**Figure 2:**
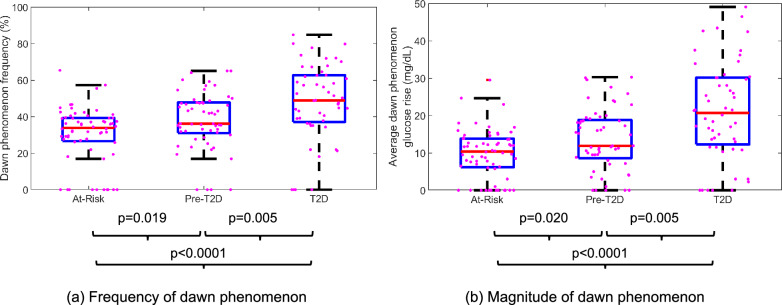
Boxplots comparing the (**a**) frequency and (**b**) average dawn phenomenon glucose rises across at-risk, pre-T2D and T2D groups in the primary cohort (n = 173) using the proposed probabilistic approach. Red horizontal line indicates the median, blue box edges represent the interquartile range, black tails represent the range of values, and magenta dots represent individual data points. P-values for pairwise comparison shown below the boxplots.

The frequency (% of days) and average magnitude (mg/dL) of the dawn phenomenon as per the probabilistic computation framework was computed for each participant and stratified by baseline HbA_1c_ into three groups: at-risk (HbA_1c_ < 5.7%), pre-T2D (5.7% ≤ HbA_1c_ ≤ 6.4%), and T2D (HbA_1c_ > 6.4%) as shown in Fig. 2. The T2D group had a significantly greater frequency of the dawn phenomenon compared to the at-risk (p < 0.0001) and pre-T2D groups (p = 0.005) (Fig. 2a). The T2D participants also had significantly larger glucose rise during the dawn phenomenon on average compared to the at-risk (p < 0.0001) and pre-T2D participants (p = 0.005) (Fig. 2b). Comparing the pre-T2D vs at-risk group, there was a higher value for both dawn phenomenon frequency (p = 0.02) and magnitude of the glucose rise (p = 0.019). This demonstrates that while the dawn phenomenon is typically associated with established T2D, the probabilistic approach using CGM suggests progressively increasing signals for the dawn phenomenon in individuals at-risk for T2D and with pre-T2D. The detailed statistical results for the frequency and magnitude of the dawn phenomenon are listed in Supplementary Table 1. Given the similar dawn phenomenon levels in the at-risk and pre-T2D groups, we performed an additional analysis comparing the probabilistic dawn phenomenon measures between a combined non-T2D group (at-risk and pre-T2D participants) and the T2D group. As expected, the T2D group had a significantly greater dawn phenomenon frequency and magnitude than the non-T2D participants (p < 0.0001) (Supplementary Table 2).

To evaluate the value added by the probabilistic approach, these results were compared with the binary model that uses a fixed 20 mg/dL threshold to estimate the dawn phenomenon. The binary model also demonstrates an increasing dawn phenomenon frequency and magnitude for the three HbA_1c_ sub-groups, as detailed in Supplementary Fig. 1 and Supplementary Table 3. However, the dawn phenomenon frequency values using the binary model were significantly lower than the probabilistic model for all three HbA_1c_ sub-groups (Supplementary Table 4), with a median 28.9, 27.6, and 23.4 percentage points greater frequency of dawn phenomenon identified by the probabilistic approach. In particular, for the at-risk cohort, the binary approach computed the median dawn phenomenon frequency to be 0% compared to an effective 34% of valid CGM days using the probabilistic approach, driven by the relatively large fraction of days with measured nocturnal glucose rise less than but close to 20 mg/dL (Fig. 1). These days are ignored by the binary approach, but contribute small probabilities on individual days using the probabilistic approach. These small daily probabilities aggregate overall to a larger effective total days of dawn phenomenon. In contrast to the dawn phenomenon frequency, the average magnitude of the dawn phenomenon glucose rise using the probabilistic approach was substantially lower for the pre-T2D and T2D groups but not for the at-risk group compared to the binary approach (Supplementary Table 4). Taken together, this finding suggests that the probabilistic model has the potential to detect the dawn phenomenon earlier in the three categories compared to the binary model.

#### Dawn phenomenon measures as an independent predictor of HbA_1c_

**Figure 3:**
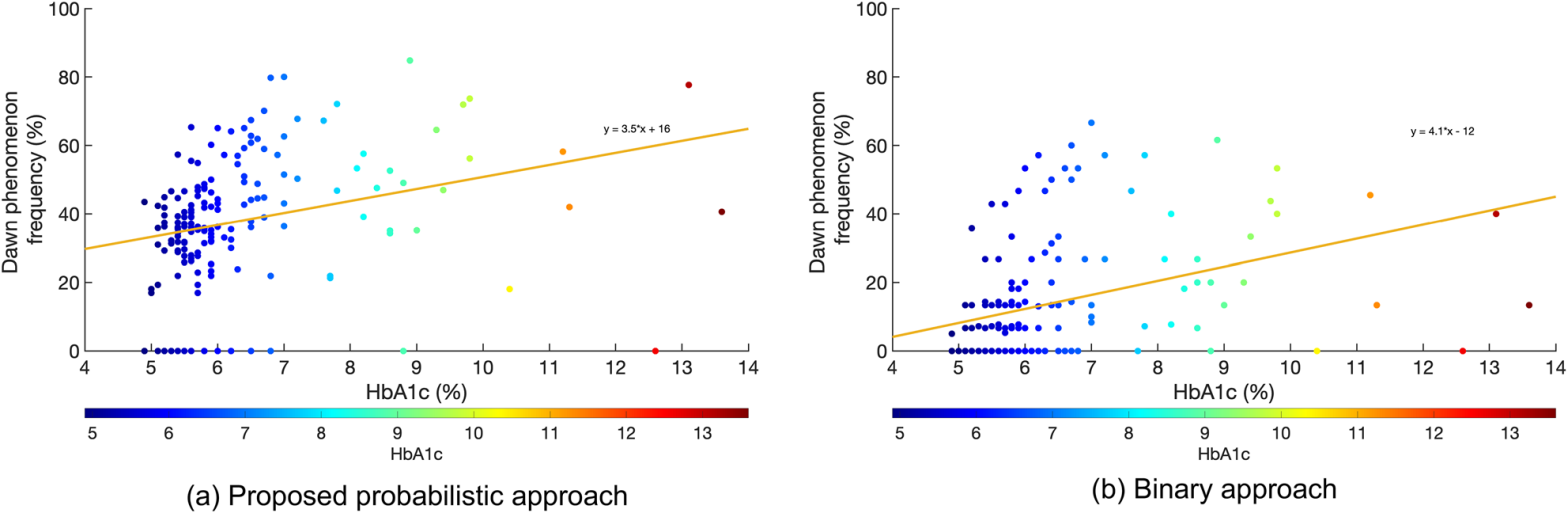
Scatter plot of HbA_1c_ vs. dawn phenomenon frequency for the primary cohort (n = 173) computed using the (**a**) proposed probabilistic approach and (**b**) binary approach respectively. Each point represents a participant, with the color of the dot representing their HbA_1c_ as outlined in the heatmap. The orange line represents the best linear fit with its equation noted on top of the line.

Subsequently, after adjusting for demographic and clinical covariates, multiple linear regression was used to examine the association between the probabilistic measures for the dawn phenomenon and HbA_1c_ (Table 2). The BMI was highly correlated with waist circumference ($\rho$=0.78, p < 0.0001) and therefore only the latter was included to avoid multicollinearity issues. As the frequency and magnitude of the dawn phenomenon were highly correlated ($\rho$=0.99, p < 0.0001), only the former was used in the model. The linear regression analysis showed that the frequency of the dawn phenomenon was significantly associated with HbA_1c_ (p = 0.00012) independently of known covariates. Waist circumference (p = 0.014) and male gender (p = 0.054) were also positively associated with HbA_1c_. None of the other covariates showed any association with the HbA_1c_. We visualize this association between the probabilistic dawn phenomenon frequency and HbA_1c_ via a scatter plot in Fig. 3a.

**Table 2:** Assessing the relationship between dawn phenomenon and the HbA_1c_.

	Estimate	Std. error	t stat	p-value
(Intercept)	3.634	1.036	3.51	0.0006***
Age	−0.009	0.009	−0.99	0.32
Gender (male)	0.470	0.242	1.95	0.054^
Waist circumference	0.023	0.009	2.49	0.014*
Hispanic/Latino	−0.648	0.423	−1.53	0.13
Born in Mexico	0.341	0.456	0.75	0.46
DP frequency	0.024	0.006	3.93	0.00012***

Multiple linear regression using the probabilistic dawn phenomenon frequency to predict HbA_1c_ after adjusting for known clinical and demographic covariates.

p-value key: ****p < 0.0001, ***p < 0.001, **p < 0.01, *p < 0.05, ^p < 0.1.

*DP* dawn phenomenon.

We next recomputed the regression model using the binary dawn phenomenon frequency measure instead (Supplementary Table 5). The binary dawn phenomenon frequency measure was significantly associated with the HbA_1c_ (p < 0.0001). Waist circumference was again associated (p = 0.007) while male gender (p = 0.064) showed a trend of association with HbA_1c_. As before, we visualize this association between the probabilistic dawn phenomenon frequency and HbA_1c_ via a scatter plot in Fig. 3b. Overall, this result shows that the probabilistic model preserves the association between dawn phenomenon and HbA_1c_ identified by the binary model after adjusting for known clinical and demographic covariates.

#### Quantifying day-to-day variations in probabilistic dawn phenomenon measures

We next quantified the effect of daily variations on the probabilistic dawn phenomenon frequency and magnitude for all participants. To do this, we computed the coefficient of variation for both measures for each participant and stratified by HbA_1c_ category (Supplementary Table 6). Participants in the T2D group had a significantly larger coefficient of variation for the dawn phenomenon frequency compared to at-risk (p = 0.02), while there were no statistical differences for either of these two groups compared to the pre-T2D group (p > 0.05). The coefficient of variation for the magnitude of nocturnal glucose rise were not statistically different across the three HbA_1c_ groups (all p > 0.05).

#### Relationship between dawn phenomenon and daytime glucose control

**Figure 4:**
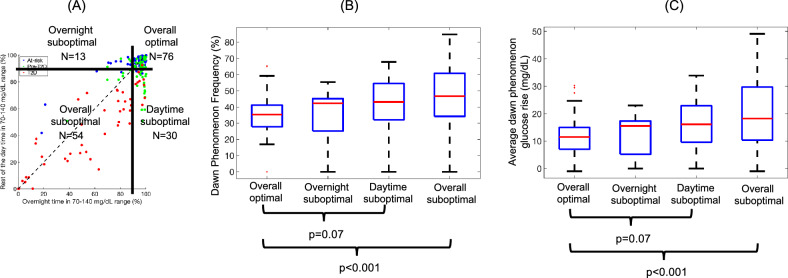
Relationship between the goodness of glucose control (measured using time in 70–140 mg/dL range [TIR_70–140_] overnight and during the day) vs. dawn phenomenon measures. (**A**) Scatterplot of overnight TIR_70-140_ vs. daytime TIR_70-140_ for n = 173 participants. Each point represents a participant colored by their HbA_1c_ classification (at-risk of T2D in blue, pre-T2D in green, and T2D in red). The black horizontal and vertical lines stratify each participant into one of four categories highlighting the goodness of their overnight and daytime glucose control. Boxplots comparing the (**B**) frequency and (**C**) magnitude of the dawn phenomenon across all participants stratified into the four TIR_70–140_ categories. Red horizontal line indicates the median, blue box edges represent the interquartile range, black tails represent the range of values, and red dots represent outliers. P-values for pairwise comparison shown below the boxplots.

The potential of probabilistic dawn phenomenon measures as early indicators of worsening glucose control was examined. Previous studies have shown an association of the dawn phenomenon with post-breakfast hyperglycemia^16^ and next-day glucose excursions^16,17^. Another study showed that increased daytime time in range might signal progression towards T2D, even when overnight glucose control is excellent^18^. Therefore, the relationship between the probabilistic method derived dawn phenomenon measures and time in 70–140 mg/dL (TIR_70-140_) was computed separately during the day (06:00 h to 00:00 h) and overnight (00:00 h to 06:00 h) following the approach outlined previously^18^. The results are demonstrated in Fig. 4. Each participant was classified into one of four groups depending on their overnight and daytime TIR_70–140_ as shown in Fig. 4a: (1) optimal overall control (both overnight and daytime TIR_70–140_ ≥ 90%); (2) sub-optimal daytime control (overnight TIR_70–140_ ≥ 90% and daytime TIR_70–140_ < 90%); (3) sub-optimal overnight control (overnight TIR_70–140_ < 90% and daytime TIR_70–140_ ≥ 90%); (4) sub-optimal overall control (both overnight and daytime TIR_70–140_ < 90%). The choice of 90% was made based on previous analysis in this cohort that showed that pre-T2D participants and at-risk participants had TIR_70–140_ of > 90% on average^18^. The frequency and magnitude of the dawn phenomenon were significantly higher for the sub-optimal overall control group than the optimal overall control group (both p < 0.001) (Fig. 4b, c). While not statistically significant, the dawn phenomenon frequency and magnitude values trended higher in the sub-optimal daytime control group vs. the optimal overall control group (p = 0.07).

#### Sensitivity analysis with varying dawn phenomenon glucose thresholds

We performed a sensitivity analysis by changing the dawn phenomenon glucose threshold of 20 mg/dL, to estimate the threshold’s impact on dawn phenomenon measures and associations with HbA_1c_. We used two cutoffs: $\gamma$=10 mg/dL^11^, which has been previously suggested as a dawn phenomenon threshold, and a symmetric higher threshold of 30 mg/dL, to assess the effect of both lowering and increasing the 20 mg/dL threshold. We first compute the dawn phenomenon likelihood functions for the three thresholds (Supplementary Fig. 2), which shows that a given measured nocturnal glucose rise has progressively lower probability of dawn phenomenon with increasing threshold. Similar to the 20 mg/dL analyses, we observed an increasing trend in the dawn phenomenon frequency and magnitude of nocturnal glucose rise with increasing HbA_1c_. This was observed for both the probabilistic (Supplementary Table 7) and binary (Supplementary Table 8) approaches. As expected, the 10 mg/dL and 30 mg/dL thresholds showed higher and lower dawn phenomenon frequency and magnitude respectively compared to the 20 mg/dL threshold. We also recomputed regression models with the 10 and 30 mg/dL thresholds for both approaches, with the dawn phenomenon frequency measure significantly associated with HbA_1c_ in both probabilistic and binary models. The 30 mg/dL threshold had a stronger association with the HbA_1c_ in our cohort compared to the 10 mg/dL threshold for both the probabilistic approach (${\beta }_{30,prob }=0.035, {SE}_{30,prob}=0.007$ vs ${\beta }_{10,prob }=0.015, S{E}_{10,prob}=0.005)$ and the binary approach (${\beta }_{30,binary }=0.045, {SE}_{30,binary}=0.018$ vs ${\beta }_{10,prob }=0.016, S{E}_{10,prob}=0.005)$ ($\beta$: regression coefficient estimate, SE: standard error of regression coefficient). Detailed results are presented in Supplementary Tables 9 and 10.

#### Generalizability of the proposed probabilistic framework to other CGM manufacturers

Further, while the dawn phenomenon calculations were based on the performance characteristics of the Freestyle Libre Pro CGM^12^, the corresponding probability distribution function can be computed for any CGM. As an example, the distribution functions of an ideal error-free CGM, a Freestyle Libre Pro CGM, and a Dexcom G6 CGM^13^ were computed as shown in Supplementary Fig. 3. Our probabilistic model can therefore be easily adapted to differences in sensor accuracy between different CGM manufacturers.

## Discussion

In this study, a new probabilistic framework to estimate the dawn phenomenon using CGM was evaluated. The proposed framework incorporates the intrinsic CGM error into the assessment of the dawn phenomenon, with the data suggesting a more robust estimation compared to previous approaches. Probabilistic modeling has the potential to detect the presence of the dawn phenomenon earlier than previous deterministic approaches that only consider days where nocturnal glucose crosses a fixed predetermined threshold. Furthermore, an observed association between probabilistic dawn phenomenon metrics and glucose control during the day could provide novel insights into links between nocturnal and diurnal hyperglycemia.

While CGMs are not yet part of routine care in individuals at risk for or with non-insulin-treated T2D, multiple studies point to their acceptability, growing use, and potential benefits in this population^18–23^. Notably, a recent study at a large medical center showed a 125% increase in CGM prescriptions in primary care setting between 2020 to 2021, with 28% of these new users not on insulin^24^. These data suggest more widespread adoption of CGM in the near future in our target cohort of individuals at risk for or with T2D not on insulin, which makes real-world implementation of our probabilistic dawn phenomenon approach more feasible. In our study, we observed a progression in the frequency and magnitude of the dawn phenomenon with increasing HbA_1c_ level, with a slight increase from at-risk to pre-T2D followed by a larger increase in dawn phenomenon measures in individuals with T2D. These findings are in line with a previous study in a cohort of Chinese adults^3^ which used a binary approach with a cut-off of 20 mg/dL. The frequency of the dawn phenomenon was 8.9% in adults with normal glucose tolerance, 30% in those with impaired glucose regulation, and 52% in individuals with a recent diagnosis of T2D. In that study, time in range between 70 and 180 mg/dL was lower and glucose variability was more common among those with a dawn phenomenon^3^. The present study provides evidence on the frequency and magnitude of the dawn phenomenon in an underserved Hispanic cohort with or at-risk for T2D. It is not known whether there are differences related to the burden and influence of the dawn phenomenon across different stages of dysglycemia between racial and ethnic groups. As such, our study provides valuable new data on the dawn phenomenon in a traditionally understudied minority population.

The probabilistic modeling of the dawn phenomenon using CGMs allowed for stratification into subgroups of individuals at-risk, with pre-T2D, and those with T2D, similar to that seen after stratification by HbA_1c_ values. In this study, the frequency and magnitude of the dawn phenomenon for individuals with T2D were significantly larger than those with pre-T2D, which in turn were larger than those for at-risk participants. The probabilistic model suggested that 37% of the effective number of dawn phenomenon days were contributed by days with measured nocturnal glucose rise of < 20 mg/dL. Thus the contribution to dawn phenomenon risk from a sizeable fraction of days is ignored by error-agnostic binary models, potentially underestimating the dawn phenomenon occurrence in this population. In addition, while the dawn phenomenon metrics computed using the binary model stratified the at-risk, pre-T2D, and T2D groups, it did so at lower median values for each category. These findings suggest that the probabilistic model has the potential to detect the onset of the dawn phenomenon *earlier* in the natural history of the progression from normoglycemia to T2D compared to the standard binary model. Early detection of the dawn phenomenon may then facilitate earlier interventions in individuals with T2D. Since there are no guidelines on dawn phenomenon therapeutic regimens for at-risk and pre-T2D populations, we do not envision our probabilistic dawn phenomenon measures as a guide for therapy in these groups. Rather, our probabilistic model may be useful as an earlier indicator for worsening dawn phenomenon as well as worsening HbA_1c_ compared to the standard binary model in individuals at-risk of T2D or with pre-T2D.

Here, multiple linear regression analysis also showed that the novel probabilistic dawn phenomenon measure was significantly associated with HbA_1c_ after adjusting for known demographic and clinical covariates. In adults with T2D, the dawn phenomenon is difficult to both prevent and treat with existing therapies. Participants in this study were predominantly Hispanic/Latino adults, a population bearing a disproportionate burden of diabetes compared to the background non-Hispanic White population^25^ while also being historically underrepresented in clinical trials^26^. It remains to be determined whether earlier detection of the dawn phenomenon using CGM in underserved populations will offer novel opportunities for innovative lifestyle as well as pharmaceutical interventions. Although basal insulin can be a useful approach, there remains significant clinical inertia in the use of insulin in T2D, although the use of CGM and other digital health technologies may help to overcome this^27^. Furthermore, while we developed our probabilistic model using CGM data in individuals with or at risk for T2D, our model can also be translated to individuals with type 1 diabetes (T1D) with modifications accounting for the amount of overnight insulin dosing in response to nocturnal glucose rise in those using automated insulin delivery systems^28,29^.

Several limitations need to be addressed in subsequent studies. The CGM profiles used represent real-world data in that participants were free-living, with no control over the amount and timing of food and physical activity. The dawn phenomenon is thought to be a consequence of an altered central circadian clock in the brain resulting in excessive hepatic glucose production during sleep and increased insulin resistance in the morning and, therefore, is less likely to be impacted by these factors. We did not have HOMA-IR and HOMA-β measures for our participants, therefore, we could not examine associations between our probabilistic dawn phenomenon measures with these known markers of insulin resistance and beta-cell function^17^. Alternatively, there is evidence that the timing and macronutrient content of meals in the evening can influence next-day glucose levels^30^. In particular, accurately timing the start of breakfast is vital to calculating the nocturnal glucose rise, and therefore the dawn phenomenon accurately. In this study, and based on previous work, a rule-based breakfast detection algorithm was applied which depends on observer assessment of CGM profiles^21^. More accurate and objective breakfast detection, through use of adaptive machine learning algorithms or additional sensors^31–33^, can improve the reliability of our findings. Another source of error is that while the Gaussian error model is a reasonable approximation for CGM measurements during the night when glucose levels are relatively stable, this approximation may not hold in non-steady state conditions^34,35^. Therefore, a Gaussian error-based probabilistic approach may not apply to individuals who experience rapid nocturnal glucose rises. Further, the current analysis did not account for sleep; a generic midnight-to-pre-breakfast period was used for all computations. Accurately measuring sleep using wearable monitors^36,37^ could help delineate the period over which to compute the nocturnal glucose nadir more accurately, which may make the dawn phenomenon analysis more precise. Although we did not expressly exclude individuals who performed shift work, which is known to impact glucose metabolism^38^, at enrollment no participants identified as shift workers. While there appeared to be an association between dawn phenomenon metrics and sub-optimal glucose control during the day in a sub-group of participants, the size of the sub-group was likely too small to detect the actual effect size. We did not compare our probabilistic dawn phenomenon model's findings against retrospective clinician evaluation, as at present there are no clear guidelines for CGM-based assessment of dawn phenomenon. We envision that our findings may serve as evidence to help formulate clinical guidelines for CGM-based dawn phenomenon determination. Finally, this study was performed in a predominantly female Hispanic/Latino cohort. Therefore, the generalizability of our dawn phenomenon results needs to be validated in cohorts with more male participants and diverse populations.

In conclusion, this study evaluated a new probabilistic model for quantifying the dawn phenomenon for individuals with or at risk for T2D. The model incorporates the intrinsic CGM measurement error leading to a more robust quantification of the dawn phenomenon. In addition, accounting for nocturnal glucose rises that are smaller than the currently used 20 mg/dL threshold allows this model to detect abnormal overnight rise in glucose levels earlier. The findings suggest a new way to quantify the dawn phenomenon using CGM, potentially enabling earlier therapeutic interventions.

## Methods

### Participant recruitment and data collection

An Independent Review Board (Advarra IRB Study 2018-01793, Protocol 00036476) reviewed all study-related protocols and approved the study. Following IRB approval and prior to participation in any activities, participants provided written informed consent to be enrolled in an observational study called Farming for Life (ClinicalTrials.gov number: NCT 03940300, registration date: January 28, 2019)^39^. All research was performed in accordance with relevant guidelines and regulations. The study consisted of two weeks of CGM wear on either side of a 10-week intervention where participants received weekly prescriptions of fresh vegetables. The Farming for Life study was conducted between February 2019 to May 2022. The complete protocol details have been published previously^18,39^. Participants were recruited via bilingual (Spanish and English) outreach materials and with help from bilingual community health workers through community outreach programs, Hispanic/Latino-focused community organizations, and local health and social services. Participants who were eligible and consented to the study protocol provided baseline demographic and clinical information on age, gender, self-reported race and ethnicity, health insurance status, and whether participants had been informed of a diagnosis of T2D by a qualified medical provider. Criteria for inclusion were adults who were 18 years of age or older, who had T2D for at least 6 months or were designated at risk for developing T2D using the American Diabetes Association (ADA) diabetes risk assessment tool^40^. Briefly, the ADA risk scoring (0–11 points) includes seven questions on age, gender, gestational diabetes mellitus (GDM), family history of diabetes, high blood pressure, physical activity and obesity status based on body mass index (BMI). A score of 5 points and higher is considered high risk of having diabetes^41^. The exclusion criteria for this study included current or previous insulin use, pregnancy, or any active clinically significant disease or disorder which could interfere with participation in the study. Guidelines from the National Health and Nutrition Examination Survey Anthropometry Procedure Manual, January 2016^42^, were used to measure height, weight, and waist circumference. The Quetelet Index (body weight in kilograms divided by height squared (meters)^43^) was used to calculate the participants’ BMI. HbA_1c_ was measured using a fingerstick meter (Siemens DCA Vantage, Siemens Healthcare, Norwood, Massachusetts, USA). Participants were stratified using HbA_1c_ into at-risk (HbA_1c_ < 5.7%), pre-T2D (5.7% ≤ HbA_1c_ ≤ 6.4%), and T2D (HbA_1c_ > 6.4%)^15^ sub-groups for data analysis. They were trained to wear blinded CGM (Abbott Freestyle Libre Pro) sensors using manufacturer educational materials under the supervision of research staff. Participants were asked to wear the CGM for 14 days after enrollment. Participants led free-living lives during these two weeks and returned to the research site for the removal of their CGM sensors at the end of the two weeks. Subsequently, the CGM reader was connected to https://www.libreview.com/ to extract timestamped glucose recordings that were used for subsequent analyses. Participants with at least ten days of CGM data per consensus guidelines^14^ were considered for the dawn phenomenon analysis.

### Probabilistic computation of the dawn phenomenon

#### Conceptual overview

**Figure 5:**
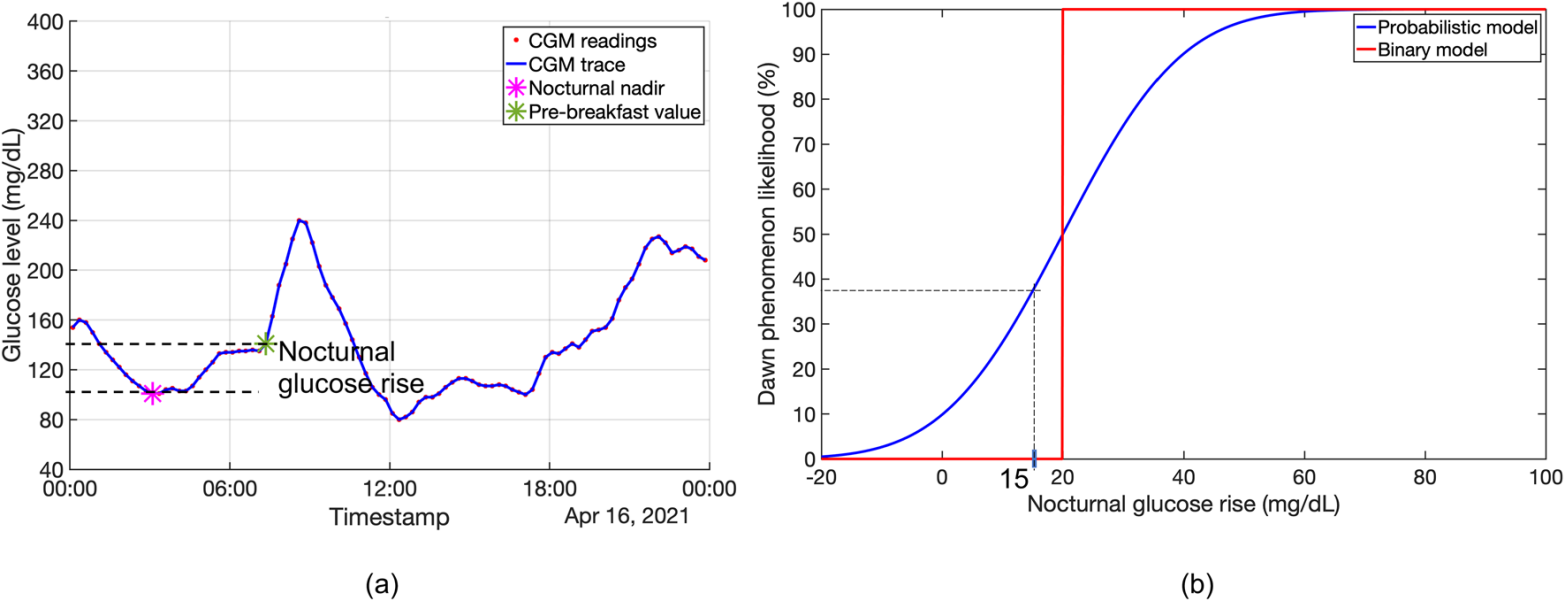
The probabilistic model to quantify the dawn phenomenon: (**a**) Computation of the nocturnal glucose rise as the difference between pre-breakfast CGM glucose reading and the nocturnal glucose nadir observed overnight. (**b**) The likelihood of dawn phenomenon for the binary and probabilistic models as a function of nocturnal glucose rise The probability of dawn phenomenon for an example nocturnal glucose rise of 15 mg/dL is different for the binary model (0%) and the probabilistic model (37%). Overall, the nocturnal glucose rise computed in (**a**) is mapped onto the probabilistic dawn phenomenon likelihood function in (**b**) to calculate the probability that the dawn phenomenon occurred that night.

Figure 5 shows the dawn phenomenon likelihood as a function of different nocturnal glucose rise values for the binary and probabilistic approaches. For the binary model, any nocturnal glucose rises below 20 mg/dL is classified as not a dawn phenomenon (or 0% likelihood of dawn phenomenon) while any value greater than or equal to 20 mg/dL is classified as a dawn phenomenon (or 100% likelihood of dawn phenomenon). In contrast, the proposed probabilistic model assigns a non-zero and increasing likelihood of dawn phenomenon for increasing nocturnal glucose rise. For example, as shown in Fig. 5(b), the error-agnostic binary model assigns a nocturnal glucose rise of 15 mg/dL with 0% likelihood of dawn phenomenon. Conversely, the probabilistic model recognizes that a reported rise of 15 mg/dL has an associated CGM device measurement error and computes a ~ 37% likelihood that the dawn phenomenon may have occurred.

The novel probabilistic framework for analyzing the dawn phenomenon is composed of four stages described below.

### I. Identification of the start of breakfast

For each day of CGM, a semi-automated framework from a previous study was used^21^ to identify the start of breakfast. The framework has a manual breakfast segment annotation stage followed by a rule-based framework to filter out non-breakfast segments. Three rules to define breakfast were applied: (i) the start time of breakfast should lie between 5 am to 11 am; (ii) the rise in glucose from breakfast start to post-breakfast peak should be equal or greater than a predefined threshold of + 40 mg/dL; (iii) if multiple segments of the glucose curve satisfied (i) and (ii), the earlier segment was designated to be the breakfast. The glucose rise threshold of + 40 mg/dL was chosen based on previous post-breakfast glucose response studies in healthy, pre-T2D, and T2D participants^44–46^.

### II. Computation of the nocturnal glucose rise

Following procedures outlined previously^3^, the nocturnal glucose nadir was calculated as the minimum CGM reading in the midnight to pre-breakfast interval. Subsequently, the nocturnal glucose rise was computed as the difference between the pre-breakfast CGM reading and the nocturnal glucose nadir. Figure 5a visually demonstrates this computation.

### III. Computation of dawn phenomenon probability for each day

A probabilistic dawn phenomenon computation framework using a Gaussian error model was created. The Gaussian error model assumes that the CGM measurement errors are independent and identically distributed (“i.i.d”) Gaussian random variables with a mean value of zero and standard deviation of $\sigma$. To calculate $\sigma$, publicly available Freestyle Libre Pro performance characteristics were used^12^. The error metrics corresponding to a glucose range of 80–180 mg/dL were used for the calculations, as this range appropriately reflects the overnight glucose levels of the participant cohort at risk for or with T2D. The error distribution was chosen to be Gaussian based on available data from the Freestyle Libre Pro accuracy manual^12^, which stated that 80.2% of Freestyle Libre Pro CGM readings lie within an error margin of $\pm$ 20 mg/dL. Based on these values, the standard deviation of each CGM measurement was estimated to be $\sigma =$ 15.6 mg/dL. Next, the nocturnal glucose rise was calculated as the difference of two Gaussian i.i.d CGM measurements and had a standard deviation of ${\sigma }_{{\text{diff}}}= \sqrt{2}\sigma =$ 21.9 mg/dL. Therefore, for a given nocturnal glucose rise on any given day, a Gaussian distribution with a mean equal to the recorded glucose rise value and a standard deviation of 21.9 mg/dL was created (Fig. 5b). Finally, using the constructed Gaussian distribution, we computed the likelihood that the dawn phenomenon occurred as the fraction of the area under the Gaussian curve greater than a given glucose rise threshold $\gamma$. The complete step-by-step probability computation framework is provided in **Appendix A**. A threshold of $\gamma$ = 20 mg/dL^6^ was used for our subsequent analyses.

We demonstrate the probabilistic dawn phenomenon computation using a simple toy example. Let us consider the scenario of 7 days of CGM wear with the following nocturnal glucose rises measured using the CGM: 10, 15, 25, 18, 12, 16, 8 (mg/dL). Using the binary approach with a 20 mg/dL threshold, we get the no. of days of dawn phenomenon = 0 + 0 + 1 + 0 + 0 + 0 + 0 = 1 day. For the proposed probabilistic approach, we determine the probability for each day from the dawn phenomenon likelihood function of Fig. 5b. We then add each day’s probability value to get the effective number of dawn phenomenon days = 0.25 + 0.37 + 0.63 + 0.45 + 0.3 + 0.4 + 0.22 = 2.6 effective days.

### IV. Summarizing the average frequency and magnitude of the dawn phenomenon for each participant

The previous step provided a probability that the dawn phenomenon had occurred each day for a given participant. The frequency of the dawn phenomenon was computed by adding the probabilities for each day that had a valid breakfast peak. Finally, the average magnitude of the dawn phenomenon for an individual was calculated by averaging the nocturnal glucose rise values over each day with a valid breakfast peak.

To compute the effective number of dawn phenomenon days (${N}_{DP}$) for the whole cohort, we used the following formula:

$${N}_{DP}=\sum_{i=1}^{D}Prob\left({d}_{i}\right),$$

where ${d}_{i}$ represents a valid day of CGM data; $Prob\left({d}_{i}\right)$ is the probability of dawn phenomenon on each day; D is the total number of valid days of CGM data. To compute the effective number of dawn phenomenon days contributed by days with nocturnal glucose rise < 20 mg/dL and ≥ 20 mg/dL separately, we simply sum $Prob\left({d}_{i}\right)$ over those sets of days separately.

### Statistical analysis

Since the dawn phenomenon analysis is part of a more extensive study, an a priori sample size calculation was not performed for the dawn phenomenon quantification tasks. Statistical analyses were performed using MATLAB (https://www.mathworks.com/, V.R2019b) and R (R Core Team (2019), https://www.R-project.org/). Comparisons across the three HbA_1c_ subgroups were made using a Kruskal–Wallis test, followed by multiple comparison testing using the Dunn’s test. Comparisons between two sub-groups were made using the Wilcoxon rank-sum test. Paired comparisons were made using the Wilcoxon signed rank test. Correlation values were computed via the Spearman rank correlation method. Multiple linear regression analyses were performed in R using its ‘lm’ function. The regression models were adjusted for potential confounders such as participant age, self-reported gender, waist circumference, whether they were of Hispanic/Latino ethnicity, and whether they were born in Mexico or not. Statistical significance cutoff was set at p = 0.05.

### Supplementary Information

Supplementary Information.

## Data Availability

The investigators agree to share de-identified individual participant data that underlie the results reported in this article, the computational and statistical analysis tools, and the study protocol with academic researchers beginning three months after publication and ending five years following article publication. Proposals should be directed to david.kerr@sutterhealth.org. To gain access, data requestors will need to sign a data access agreement.

## Code availability

Request for the MATLAB and R codes used to generate the figures and tables should be directed to Souptik.Barua@nyulangone.org or ashu@rice.edu.

## References

[1] Schmidt MI, Hadji-Georgopoulos A, Rendell M, Margolis S, Kowarski A (1981). The Dawn phenomenon, an early morning glucose rise: Implications for diabetic intraday blood glucose variation. Diabetes Care.

[2] Bolli GB, Gerich JE (1984). The ‘dawn phenomenon’—A common occurrence in both non-insulin-dependent and insulin-dependent diabetes mellitus. N. Engl. J. Med..

[3] Li C (2020). The dawn phenomenon across the glycemic continuum: Implications for defining dysglycemia. Diabetes Res. Clin. Pract..

[4] Kruszynska YT, Home PD (1988). Night-time metabolic changes in normal subjects in the absence of the dawn phenomenon. Diabete Metab..

[5] Schmidt MI, Lin QX, Gwynne JT, Jacobs S (1984). Fasting early morning rise in peripheral insulin: evidence of the dawn phenomenon in nondiabetes. Diabetes Care.

[6] Monnier L, Colette C, Dejager S, Owens D (2013). Magnitude of the dawn phenomenon and its impact on the overall glucose exposure in type 2 diabetes: is this of concern?. Diabetes Care.

[7] Carroll MF, Schade DS (2005). The dawn phenomenon revisited: Implications for diabetes therapy. Endocrine Pract..

[8] Monnier L, Lapinski H, Colette C (2003). Contributions of fasting and postprandial plasma glucose increments to the overall diurnal hyperglycemia of type 2 diabetic patients. Diabetes Care.

[9] Monnier L (2012). Frequency and severity of the dawn phenomenon in type 2 diabetes: Relationship to age. Diabetes Care.

[10] Monnier L, Colette C, Dejager S, Owens D (2015). The dawn phenomenon in type 2 diabetes: How to assess it in clinical practice?. Diabetes Metab..

[11] Bouchonville MF, Jaghab JJ, Duran-Valdez E, Schrader RM, Schade DS (2014). The effectiveness and risks of programming an insulin pump to counteract the dawn phenomenon in type 1 diabetes. Endocr. Pract..

[12] Abbott. *Abbott Freestyle Libre Performance Manual*.

[13] Dexcom. *Dexcom G6 Performance Manual*.

[14] Bergenstal, R. M. Understanding continuous glucose monitoring data. In *Role of Continuous Glucose Monitoring in Diabetes Treatment* (American Diabetes Association, 2018). 34251769

[15] Association AD (2021). 2. Classification and diagnosis of diabetes: Standards of medical care in diabetes—2021. Diabetes Care.

[16] King AB, Clark D, Wolfe GS (2012). Contribution of the dawn phenomenon to the fasting and postbreakfast hyperglycemia in type 1 diabetes treated with once-nightly insulin glargine. Endocr. Pract..

[17] Wang J-S (2021). The dawn phenomenon in type 2 diabetes: Its association with glucose excursions and changes after oral glucose-lowering drugs. Ther. Adv. Chronic Dis..

[18] Barua S (2021). Dysglycemia in adults at risk for or living with non-insulin treated type 2 diabetes: Insights from continuous glucose monitoring. EClinicalMedicine.

[19] Ben-Yacov O (2021). Personalized postprandial glucose response-targeting diet versus mediterranean diet for glycemic control in prediabetes. Diabetes Care.

[20] Rein M (2022). Effects of personalized diets by prediction of glycemic responses on glycemic control and metabolic health in newly diagnosed T2DM: A randomized dietary intervention pilot trial. BMC Med..

[21] Barua S (2022). The northeast glucose drift: Stratification of post-breakfast dysglycemia among predominantly Hispanic/Latino adults at-risk or with type 2 diabetes. eClinicalMedicine.

[22] Moon SJ (2023). Efficacy of intermittent short-term use of a real-time continuous glucose monitoring system in non-insulin-treated patients with type 2 diabetes: A randomized controlled trial. Diabetes Obes. Metab..

[23] Dabbagh Z (2022). The expanding use of continuous glucose monitoring in type 2 diabetes. Diabetes Technol. Ther..

[24] Mayberry LS, Guy C, Hendrickson CD, McCoy AB, Elasy T (2023). Rates and correlates of uptake of continuous glucose monitors among adults with type 2 diabetes in primary care and endocrinology settings. J. Gen. Intern. Med..

[25] Morales J (2020). Understanding the impact of five major determinants of health (genetics, biology, behavior, psychology, society/environment) on type 2 diabetes in US Hispanic/Latino families: Mil Familias—A cohort study. BMC Endocr. Disord..

[26] Occa A, Morgan SE, Potter JE (2018). Underrepresentation of Hispanics and other minorities in clinical trials: Recruiters’ perspectives. J. Racial Ethnic Health Disparities.

[27] Kerr D, Edelman S, Vespasiani G, Khunti K (2022). New digital health technologies for insulin initiation and optimization for people with type 2 diabetes. Endocr. Pract..

[28] Perriello G (1991). The dawn phenomenon in type 1 (insulin-dependent) diabetes mellitus: Magnitude, frequency, variability, and dependency on glucose counterregulation and insulin sensitivity. Diabetologia.

[29] Lindmeyer AM, Meier JJ, Nauck MA (2020). Patients with type 1 diabetes treated with insulin pumps need widely heterogeneous basal rate profiles ranging from negligible to pronounced diurnal variability. J. Diabetes Sci. Technol..

[30] Abbie E, Francois ME, Chang CR, Barry JC, Little JP (2020). A low-carbohydrate protein-rich bedtime snack to control fasting and nocturnal glucose in type 2 diabetes: A randomized trial. Clin. Nutr..

[31] Sharma S, Hoover A (2022). Top-down detection of eating episodes by analyzing large windows of wrist motion using a convolutional neural network. Bioengineering.

[32] Doulah A (2017). Meal microstructure characterization from sensor-based food intake detection. Front. Nutr..

[33] Popp CJ (2023). Objective determination of eating occasion timing (OREO): Combining self-report, wrist motion, and continuous glucose monitoring to detect eating occasions in adults with pre-diabetes and obesity. J. Diabetes Sci. Technol..

[34] Pleus S (2015). Rate-of-change dependence of the performance of two CGM systems during induced glucose swings. J. Diabetes Sci. Technol..

[35] Barua S, Wierzchowska-McNew R, Deutz NEP, Sabharwal A (2022). Discordance between postprandial plasma glucose measurement and continuous glucose monitoring. Am. J. Clin. Nutr..

[36] de Zambotti M, Cellini N, Goldstone A, Colrain IM, Baker FC (2019). Wearable sleep technology in clinical and research settings. Med. Sci. Sports Exerc..

[37] Lee J-M, Byun W, Keill A, Dinkel D, Seo Y (2018). Comparison of wearable trackers’ ability to estimate sleep. Int. J. Environ. Res. Public Health.

[38] Hemmer A (2021). The effects of shift work on cardio-metabolic diseases and eating patterns. Nutrients.

[39] Kerr D (2020). Farming for life: Impact of medical prescriptions for fresh vegetables on cardiometabolic health for adults with or at risk of type 2 diabetes in a predominantly Mexican–American population. BMJ Nutr. Prevent. Health.

[40] Bang H (2009). Development and validation of a patient self-assessment score for diabetes risk. Ann. Intern. Med..

[41] Mohd Fauzi, N. F., Wafa, S. W., Mohd Ibrahim, A., Bhaskar Raj, N. & Nurulhuda, M. H. Translation and validation of American Diabetes Association diabetes risk test: The Malay version. *Malays J. Med. Sci.* **29**, 113–125 (2022). 10.21315/mjms2022.29.1.11 PMC8887991 35283673

[42] *NHANES 2017–2018 Procedure Manuals*. https://wwwn.cdc.gov/nchs/nhanes/continuousnhanes/manuals.aspx?BeginYear=2017.

[43] WHO Expert Committee on Physical Status . *The Use and Interpretation of Anthropometry (1993 : Geneva, S. & Organization, W. H. Physical Status : The Use of and Interpretation of Anthropometry, Report of a WHO Expert Committee*. (World Health Organization, 1995). 8594834

[44] Kanamori K (2017). Postprandial glucose surges after extremely low carbohydrate diet in healthy adults. Tohoku J. Exp. Med..

[45] Mustad VA (2020). Use of a diabetes-specific nutritional shake to replace a daily breakfast and afternoon snack improves glycemic responses assessed by continuous glucose monitoring in people with type 2 diabetes: A randomized clinical pilot study. BMJ Open Diabetes Res. Care.

[46] Bittel AJ (2021). A single bout of premeal resistance exercise improves postprandial glucose metabolism in obese men with prediabetes. Med. Sci. Sports Exerc..

