# Supplementary material for: A probabilistic computation framework to estimate the dawn phenomenon in type 2 diabetes using continuous glucose monitoring
Source: Sci Rep. 2024 Feb 5;14:2915. doi: 10.1038/s41598-024-52461-1 (PMC10844336; doi:10.1038/s41598-024-52461-1)
Supplement: Supplementary file 1 — Supplementary Information. [file 41598_2024_52461_MOESM1_ESM.docx]

**SUPPLEMENTARY DATA**

**A Probabilistic Computation Framework to Estimate the Dawn Phenomenon in Type 2 Diabetes Using Continuous Glucose Monitoring**

Souptik Barua, Namino Glantz, Arianna Larez, Wendy Bevier, Ashutosh Sabharwal and David Kerr

**Supplementary Table 1: Detailed statistical results for the dawn phenomenon frequency and magnitude for each HbA_1c_ subgroup in the primary cohort (n=173) using the proposed probabilistic model. Glucose threshold to define dawn phenomenon is 20 mg/dL.**

| **Dawn phenomenon measure** | **At-risk of T2D HbA_1c_ < 5.7%**  **(n=64)** | **Pre-T2D 5.7%<=HbA_1c_<=6.4%**  **(n=57)** | **T2D HbA_1c_>6.4%**  **(n=52)** | **KW p-value** |
| --- | --- | --- | --- | --- |
| Dawn phenomenon frequency (% of days) | 33.8 [26.5,39.2] | 36.1 [31.0,47.7] | 48.9 [37.0,62.7] | <0.0001 |
| Dawn phenomenon magnitude (mg/dL) | 10.4 [6.2,13.8] | 11.9 [8.6,18.8] | 20.7 [12.3,30.1] | <0.0001 |
| Values reported as median [interquartile range]. T2D: Type 2 diabetes, KW: Kruskal Wallis 3-way test | | | | |

**Supplementary Table 2: Detailed statistical results for the dawn phenomenon frequency and magnitude for non-T2D vs T2D subgroups in the primary cohort (n=173) using the proposed probabilistic model. Glucose threshold to define dawn phenomenon is 20 mg/dL.**

| **Dawn phenomenon measure** | **Non-T2D HbA_1c_ <= 6.4%**  **(n=121)** | **T2D HbA_1c_>6.4%**  **(n=52)** | **Wilcoxon RSM test**  **p-value** |
| --- | --- | --- | --- |
| Dawn phenomenon frequency (% of days) | 35.7 [27.7, 43.3] | 48.9 [37.0,62.7] | <0.0001 |
| Dawn phenomenon magnitude (mg/dL) | 11.6 [7.0,16.3] | 20.7 [12.3,30.1] | <0.0001 |
| Values reported as median [interquartile range]. T2D: Type 2 diabetes, Wilcoxon RSM test: Wilcoxon rank-sum test | | | |

**Supplementary Table 3: Detailed statistical results for the dawn phenomenon frequency and magnitude for each HbA_1c_ subgroup in the primary cohort (n=173) using the binary model. Glucose threshold to define dawn phenomenon is 20 mg/dL.**

| **Dawn phenomenon measure** | **At-risk of T2D HbA_1c_ < 5.7%**  **(n=64)** | **Pre-T2D 5.7%<=HbA_1c_<=6.4%**  **(n=57)** | **T2D HbA_1c_>6.4%**  **(n=52)** | **KW p-value** |
| --- | --- | --- | --- | --- |
| Dawn phenomenon frequency (% of days) | 0.0 [0.0,6.7] | 6.7 [0.0,20.0] | 20.0 [7.4,44.6] | <0.0001 |
| Dawn phenomenon magnitude (mg/dL) | 0.0 [0.0,23.5] | 24.0 [0.0,32.0] | 31.9 [23.5,39.8] | <0.0001 |
| Values reported as median [interquartile range]. T2D: Type 2 diabetes, KW: Kruskal Wallis 3-way test. | | | | |

**Supplementary Table 4: Comparison of the dawn phenomenon frequency and magnitude for each HbA_1c_ subgroup in the primary cohort (n=173) between the binary model and the proposed probabilistic model. Glucose threshold to define dawn phenomenon is 20 mg/dL.**

| **Dawn phenomenon measure** | **Difference in Dawn phenomenon measures between the binary model and proposed probabilistic model compared using a Wilcoxon signed-rank test** | | | |
| --- | --- | --- | --- | --- |
|  | **At-risk of T2D HbA_1c_ < 5.7%**  **(n=64)** | **Pre-T2D 5.7%<=HbA_1c_<=6.4%**  **(n=57)** | **T2D HbA_1c_>6.4%**  **(n=52)** | **Overall cohort**  **(n=173)** |
| Dawn phenomenon frequency (% of days) | 28.9 [20.6,33.4]  (p<0.0001) | 27.6 [20.9,31.9]  (p<0.0001) | 23.4 [13.5,30.7]  (p<0.0001) | 27.2 [18.0,32.1]  (p<0.0001) |
| Dawn phenomenon magnitude (mg/dL) | 0.0 [-10.1,7.7]  (p=0.33) | -6.1 [-13.2,3.1]  (p=0.0009) | -7.1 [-12.9,-2.9]  (p<0.0001) | -5.4 [-11.0,2.4]  (p<0.0001) |
| Values reported as median [interquartile range]. A positive value indicates the corresponding dawn phenomenon measure was higher in the probabilistic model compared to the binary model. T2D: Type 2 diabetes. | | | | |

**Supplementary Table 5. Multiple linear regression model to quantify association between dawn phenomenon frequency computed using the binary approach and HbA_1c_ . Glucose threshold to define dawn phenomenon is 20 mg/dL. Model was adjusted for known demographic and clinical predictors as listed below.**

| **Predictor** | **Estimate** | **Std. Error** | **t stat** | **p-value** |
| --- | --- | --- | --- | --- |
| **(Intercept)** | 4.16 | 0.99 | 4.18 | <0.0001**** |
| **Age** | -0.013 | 0.009 | -1.39 | 0.17 |
| **Gender (Male)** | 0.43 | 0.23 | 1.86 | 0.064^ |
| **Waist circumference** | 0.024 | 0.009 | 2.73 | 0.007** |
| **Hispanic/Latino** | -0.97 | 0.41 | -2.39 | 0.018* |
| **Born in Mexico** | 0.57 | 0.44 | 1.29 | 0.20 |
| **Dawn phenomenon frequency** | 0.034 | 0.006 | 5.41 | <0.0001**** |
| **p-value key: ****p<0.0001, ***p<0.001, **p<0.01,*p<0.05,^p<0.1** | | | | |

**Supplementary Table 6: Detailed statistical results for the coefficient of variation for the dawn phenomenon frequency for each HbA_1c_ subgroup in the primary cohort (n=173) using the probabilistic model. Glucose threshold to define dawn phenomenon is 20 mg/dL.**

| **Dawn phenomenon measure** | **At-risk of T2D HbA_1c_ < 5.7%**  **(n=64)** | **Pre-T2D 5.7%<=HbA_1c_<=6.4%**  **(n=57)** | **T2D HbA_1c_>6.4%**  **(n=52)** | **KW p-value** |
| --- | --- | --- | --- | --- |
| Coefficient of variation for Dawn phenomenon frequency (%) | 27.0 [0.0, 39.3] | 30.5 [21.8, 41.9] | 36.1 [26.1,50.7] | 0.049 |
| Coefficient of variation for Dawn phenomenon magnitude (%) | 46.6 [0.0, 74. 1] | 52.1 [32.8, 81.2] | 56.5 [38. 5,87.0] | 0.22 |
| Values reported as median [interquartile range]. T2D: Type 2 diabetes, KW: Kruskal Wallis 3-way test. | | | | |

**Supplementary Table 7: Sensitivity analysis for the dawn phenomenon frequency and magnitude for each HbA_1c_ subgroup in the primary cohort (n=173) using the proposed probabilistic model. Thresholds for dawn phenomenon used are 10 and 30 mg/dL.**

| **Dawn phenomenon measure** | **At-risk of T2D HbA_1c_ < 5.7%**  **(n=64)** | **Pre-T2D 5.7%<=HbA_1c_<=6.4%**  **(n=57)** | **T2D HbA_1c_>6.4%**  **(n=52)** | **KW p-value** |
| --- | --- | --- | --- | --- |
| **Dawn phenomenon threshold = 10 mg/dL** | | | | |
| Dawn phenomenon frequency (% of days) | 50.6 [43.1, 56.6] | 53.1 [47.5,64.3] | 64.1 [52.4,75.7] | <0.0001 |
| Dawn phenomenon magnitude (mg/dL) | 10.4 [6.2,13.8] | 11.9 [8.6,18.8] | 20.7 [12.3,30.1] | <0.0001 |
| **Dawn phenomenon threshold = 30 mg/dL** | | | | |
| Dawn phenomenon frequency (% of days) | 19.8 [14.0,23.9] | 22.3 [17.6,32.2] | 34.6 [22.9,48.4] | <0.0001 |
| Dawn phenomenon magnitude (mg/dL) | 10.4 [6.2,13.8] | 11.9 [8.6,18.8] | 20.7 [12.3,30.1] | <0.0001 |
| Values reported as median [interquartile range]. T2D: Type 2 diabetes, KW: Kruskal Wallis 3-way test. The Dawn phenomenon magnitudes are equal regardless of threshold because we define it as the average nocturnal glucose rise over all valid days of CGM data and is therefore independent of the threshold. | | | | |

**Supplementary Table 8: Sensitivity analysis for the dawn phenomenon frequency and magnitude for each HbA_1c_ subgroup in the primary cohort (n=173) using the binary model. Thresholds for dawn phenomenon used are 10 and 30 mg/dL.**

| **Dawn phenomenon measure** | **At-risk of T2D HbA_1c_ < 5.7%**  **(n=64)** | **Pre-T2D 5.7%<=HbA_1c_<=6.4%**  **(n=57)** | **T2D HbA_1c_>6.4%**  **(n=52)** | **KW p-value** |
| --- | --- | --- | --- | --- |
| **Dawn phenomenon threshold = 10 mg/dL** | | | | |
| Dawn phenomenon frequency (% of days) | 10.0 [0.0,20.0] | 21.7 [7.1,46.7] | 32.1 [17.1, 53.3] | <0.0001 |
| Dawn phenomenon magnitude (mg/dL) | 15.6 [0.0,20.1] | 19.0 [12.4,24.2] | 27.1 [20.3,36.6] | <0.0001 |
| **Dawn phenomenon threshold = 30 mg/dL** | | | | |
| Dawn phenomenon frequency (% of days) | 0.0 [0.0,0.0] | 0.0 [0.0,6.7] | 8.1 [0.0,27.0] | <0.0001 |
| Dawn phenomenon magnitude (mg/dL) | 0.0 [0.0,0.0] | 0.0 [0.0,34.9] | 36.2 [0.0,46.8] | <0.0001 |
| Values reported as median [interquartile range]. T2D: Type 2 diabetes, KW: Kruskal Wallis 3-way test. The dawn phenomenon magnitudes change with the threshold in the binary model because we define it as the average nocturnal glucose rise over only those days on which the threshold is exceeded. | | | | |

**Supplementary Table 9. Multiple linear regression to quantify association between dawn phenomenon frequency computed using the proposed probabilistic approach and HbA_1c_. Dawn phenomenon glucose thresholds used are 10 and 30 mg/dL. Both models adjusted for known demographic and clinical predictors as listed below.**

| **Regression results for Dawn phenomenon glucose threshold = 10 mg/dL** | | | | |
| --- | --- | --- | --- | --- |
| **Predictor** | **Estimate** | **Std. Error** | **t stat** | **p-value** |
| **(Intercept)** | 3.6 | 1.07 | 3.34 | 0.0011** |
| **Age** | -0.007 | 0.01 | -0.69 | 0.49 |
| **Gender (Male)** | 0.47 | 0.25 | 1.91 | 0.058^ |
| **Waist circumference** | 0.023 | 0.009 | 2.48 | 0.014* |
| **Hispanic/Latino** | -0.67 | 0.43 | -1.54 | 0.13 |
| **Born in Mexico** | 0.34 | 0.47 | 0.73 | 0.47 |
| **Dawn phenomenon frequency** | 0.015 | 0.005 | 2.83 | 0.005** |
| **Regression results for Dawn phenomenon glucose threshold = 30 mg/dL** | | | | |
| **Predictor** | **Estimate** | **Std. Error** | **t stat** | **p-value** |
| **(Intercept)** | 3.8 | 1.006 | 3.79 | 0.0002*** |
| **Age** | -0.01 | 0.009 | -1.16 | 0.25 |
| **Gender (Male)** | 0.48 | 0.236 | 2.01 | 0.046* |
| **Waist circumference** | 0.02 | 0.009 | 2.45 | 0.015* |
| **Hispanic/Latino** | -0.65 | 0.412 | -1.59 | 0.11 |
| **Born in Mexico** | 0.35 | 0.445 | 0.78 | 0.43 |
| **Dawn phenomenon frequency** | 0.035 | 0.007 | 4.97 | <0.0001**** |
| **p-value key: ****p<0.0001, ***p<0.001, **p<0.01,*p<0.05,^p<0.1.** | | | | |

**Supplementary Table 10. Multiple linear regression model to quantify association between dawn phenomenon frequency computed using the binary approach and HbA_1c_ . Dawn phenomenon glucose thresholds used are 10 and 30 mg/dL. Both models adjusted for known demographic and clinical predictors as listed below.**

| **Regression results for Dawn phenomenon glucose threshold = 10 mg/dL** | | | | |
| --- | --- | --- | --- | --- |
| **Predictor** | **Estimate** | **Std. Error** | **t stat** | **p-value** |
| **(Intercept)** | 3.9 | 1.05 | 3.73 | 0.0002*** |
| **Age** | -0.008 | 0.010 | -0.82 | 0.41 |
| **Gender (Male)** | 0.47 | 0.25 | 1.92 | 0.057^ |
| **Waist circumference** | 0.025 | 0.009 | 2.62 | 0.0096** |
| **Hispanic/Latino** | -0.84 | 0.43 | -1.96 | 0.052^ |
| **Born in Mexico** | 0.46 | 0.46 | 0.99 | 0.32 |
| **Dawn phenomenon frequency** | 0.016 | 0.005 | 3.17 | 0.002** |
| **Regression results for Dawn phenomenon glucose threshold = 30 mg/dL** | | | | |
| **Predictor** | **Estimate** | **Std. Error** | **t stat** | **p-value** |
| **(Intercept)** | 4.12 | 0.98 | 4.19 | <0.0001**** |
| **Age** | -0.007 | 0.009 | -0.77 | 0.44 |
| **Gender (Male)** | 0.53 | 0.23 | 2.30 | 0.023* |
| **Waist circumference** | 0.022 | 0.009 | 2.54 | 0.012* |
| **Hispanic/Latino** | -0.79 | 0.40 | -1.98 | 0.0498* |
| **Born in Mexico** | 0.484 | 0.43 | 1.11 | 0.27 |
| **Dawn phenomenon frequency** | 0.045 | 0.008 | 5.84 | <0.0001**** |
| **p-value key: ****p<0.0001, ***p<0.001, **p<0.01,*p<0.05,^p<0.1.** | | | | |

**Supplementary Figure 1: Boxplots comparing the (a) frequency and (b) average dawn phenomenon glucose rises across at-risk, pre-T2D and T2D groups in the primary cohort (n=173) using the binary model. Red horizontal line indicates the median, blue box edges represent the interquartile range, black tails represent the range of values, and red dots represent outliers. P-values for pairwise comparison shown below the boxplots. Magenta dots represent individual data points. Dawn phenomenon glucose threshold used is 20 mg/dL.**

**
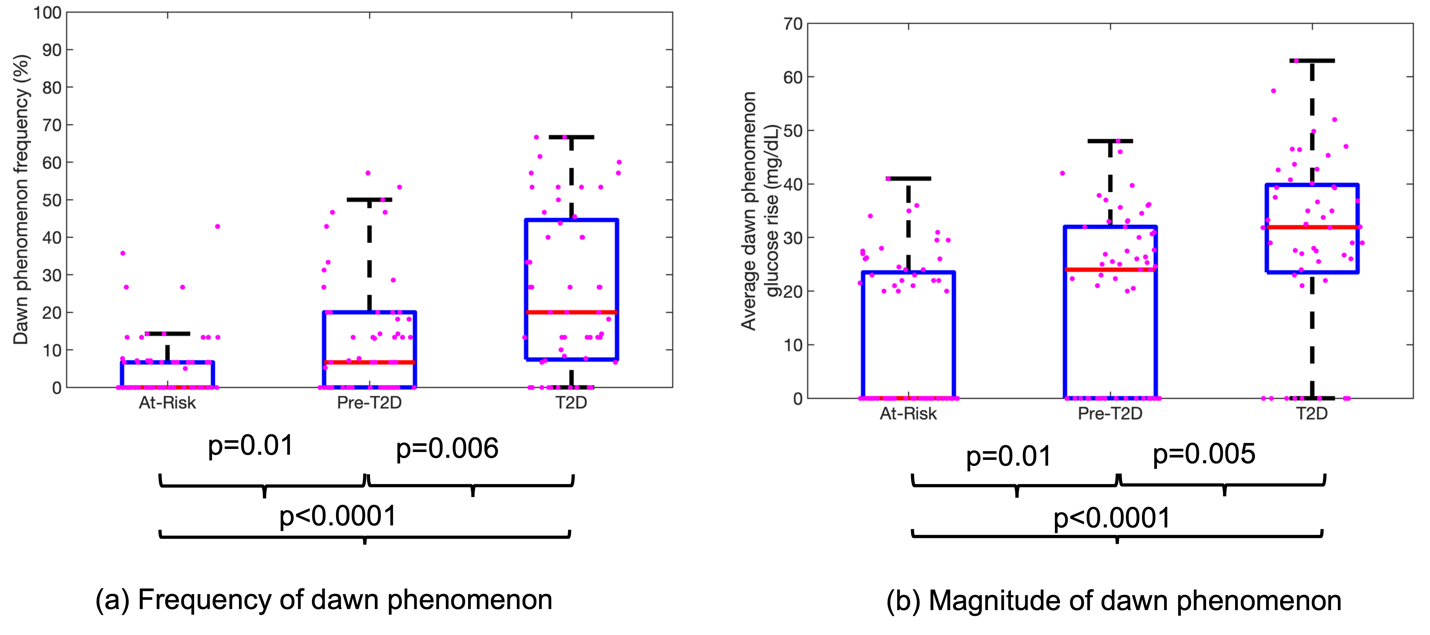
**

**Supplementary Figure 2: Dawn phenomenon likelihood functions for a given nocturnal glucose rise for two different glucose thresholds of 10 mg/dL (blue) and 20 mg/dL (red). The measured nocturnal glucose rise is on the x-axis and the corresponding dawn phenomenon likelihood for those glucose rises is on the y-axis. The proposed probabilistic framework can be generalized to any glucose threshold used to detect the dawn phenomenon.**


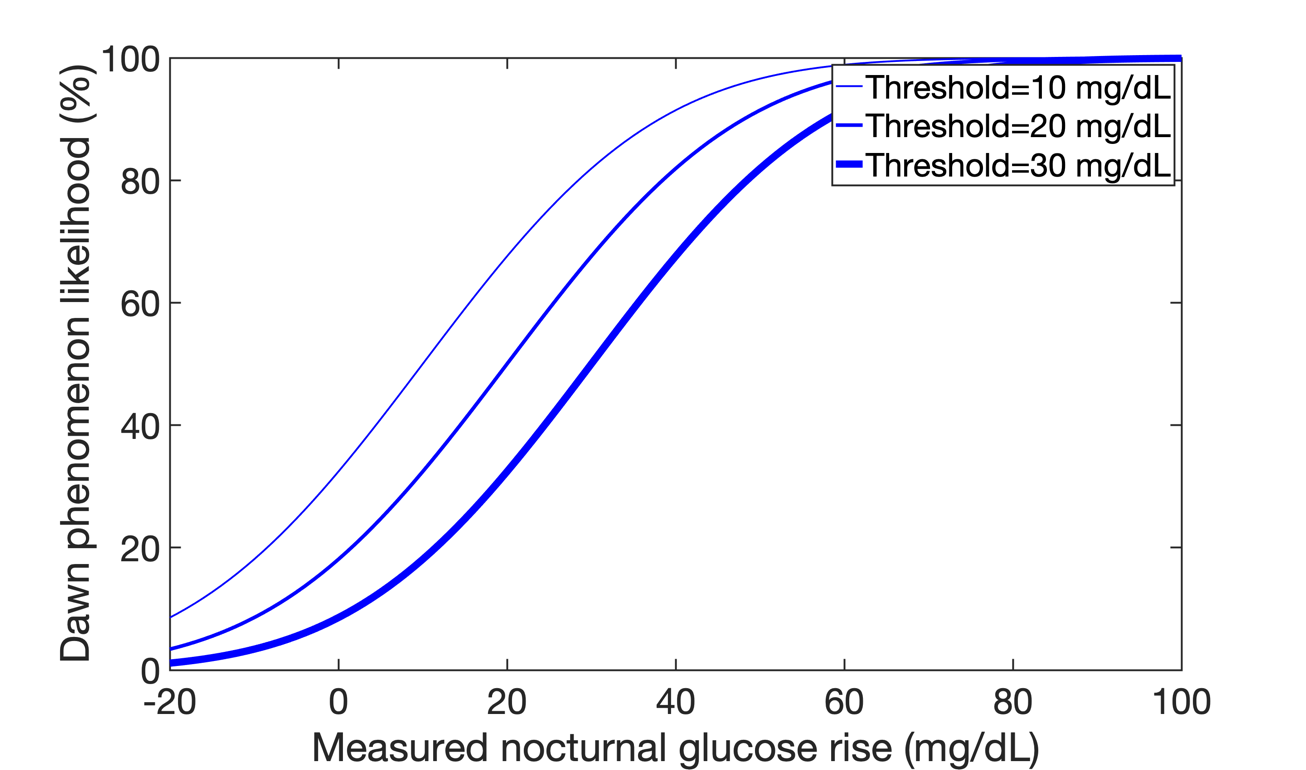


**Supplementary Figure 3: Dawn phenomenon likelihood functions for an ideal error-free sensor (red), the Freestyle Libre Pro (blue), and the Dexcom G6 (green) based on their published performance characteristics. The measured nocturnal glucose rise is on the x-axis and the corresponding dawn phenomenon likelihood for those glucose rises is on the y-axis. The proposed probabilistic framework can be adapted to any CGM device provided their measurement error statistics are known.**


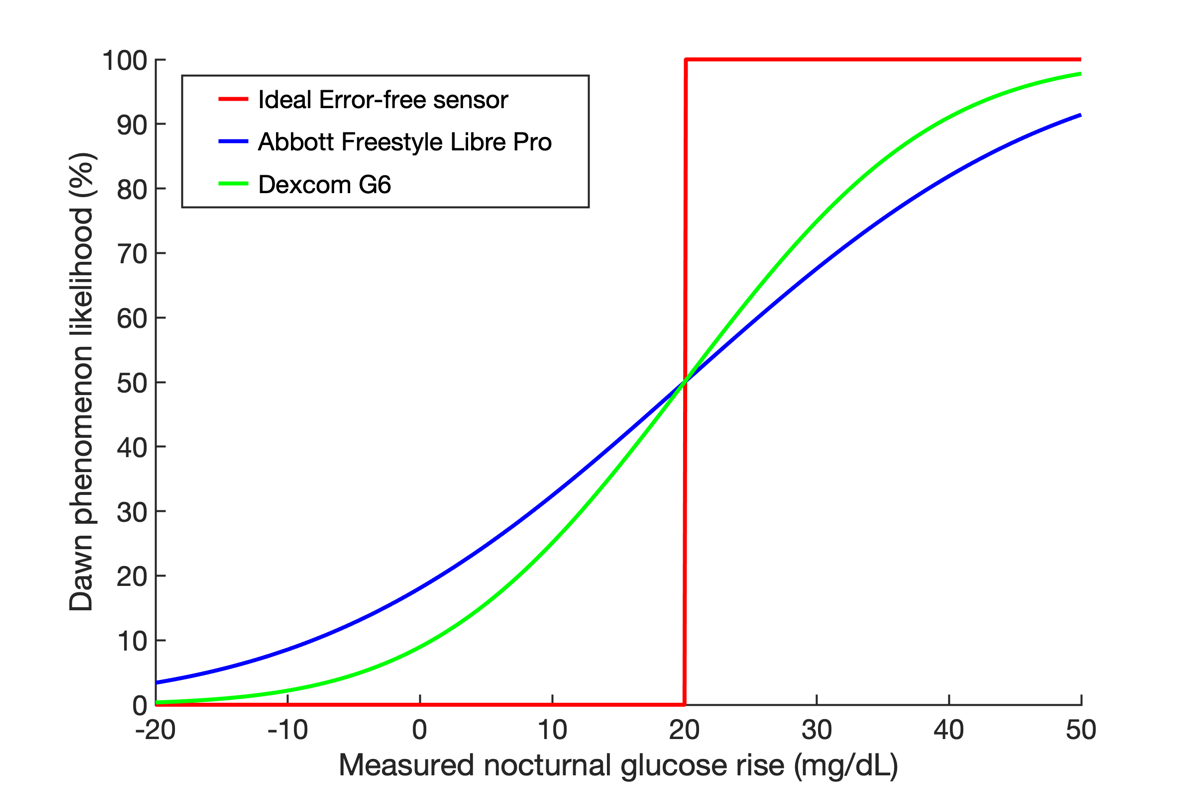


**APPENDIX A**

Mathematical formulation of the dawn phenomenon probability computation framework

The nocturnal glucose rise is defined as the difference between the nocturnal glucose nadir and the pre-breakfast glucose value. Depending on the value of this difference, we want to estimate the likelihood that the dawn phenomenon has occurred; in other words, we would like to compute the probability that the nocturnal glucose rise is greater than 20 mg/dL.

We start with the equation for the nocturnal glucose rise

$$\Delta_{diff}=x_{bfast}-x_{min};$$

Where $\Delta_{diff}$is the nocturnal glucose rise; $x_{bfast}$ and $x_{min}$ are respectively the pre-breakfast glucose and the nocturnal glucose nadir.

Including the measurement error, this equation can be rewritten as:

$\Delta_{diff}={{(x}^{0}}_{bfast}+\epsilon_{bfast} )-{(x^{0}}_{min}+ \epsilon_{min}$)

Where $\epsilon_{bfast}$ and $\epsilon_{min}$ denote the measurement error for these two CGM readings.

Rearranging the terms, we have:

$\Delta_{diff}={{(x}^{0}}_{bfast}-{x^{0}}_{min})+(\epsilon_{bfast} - \epsilon_{min}$)

Since the measurement errors are Gaussian random variables, $\Delta_{diff}$ is a Gaussian random variable with mean $\mu_{diff}$and standard deviation (s.d.) $\sigma_{diff}$ where
$\mu_{diff}$= ${{(x}^{0}}_{bfast}-{x^{0}}_{min})$ is the expected value of the nocturnal glucose rise

$\sigma_{diff}= {\sqrt{{\sigma^{2}}_{bfast}+ {\sigma^{2}}_{min}}}= \sqrt{2} \sigma_{CGM}$ is the s.d of the difference of two Gaussian i.i.d random variables with individual s.d.’s of $\sigma_{CGM}$.

Using Abbott’s performance manual^12^, 80% of the Abbott Freestyle Libre Pro’s readings (in the range 70-180 mg/dL range) lie within $\pm$20% of the true value. Using this information, we can calculate $\sigma_{CGM}$= 15.6 mg/dL and therefore $\sigma_{diff}= \sqrt{2} \times$ 15.6 = 21.9 mg/dL.

We model the likelihood of dawn phenomenon for a given value of nocturnal glucose rise by constructing a Gaussian centered on that nocturnal rise value with standard deviation of 21.9 mg/dL, and then computing the z-score at 20 mg/dL.
